# Extrinsic neuromodulation in the rodent olfactory bulb

**DOI:** 10.1007/s00441-020-03365-9

**Published:** 2020-12-23

**Authors:** Daniela Brunert, Markus Rothermel

**Affiliations:** Department of Chemosensation, AG Neuromodulation, Institute for Biology II, RWTH Aachen University, 52074 Aachen, Germany

**Keywords:** Olfactory bulb, Neuromodulation, Rodents, Olfactory processing, Perception

## Abstract

Evolutionarily, olfaction is one of the oldest senses and pivotal for an individual’s health and survival. The olfactory bulb (OB), as the first olfactory relay station in the brain, is known to heavily process sensory information. To adapt to an animal’s needs, OB activity can be influenced by many factors either from within (intrinsic neuromodulation) or outside (extrinsic neuromodulation) the OB which include neurotransmitters, neuromodulators, hormones, and neuropeptides. Extrinsic sources seem to be of special importance as the OB receives massive efferent input from numerous brain centers even outweighing the sensory input from the nose. Here, we review neuromodulatory processes in the rodent OB from such extrinsic sources. We will discuss extrinsic neuromodulation according to points of origin, receptors involved, affected circuits, and changes in behavior. In the end, we give a brief outlook on potential future directions in research on neuromodulation in the OB.

## Introduction

We live in an ever-changing environment that poses an enormous challenge to our nervous system. Granting our behavioral flexibility, the ability to perceive and act upon sensory stimuli in a differentiated way is a process called neuromodulation. Neuromodulation, defined as “the alteration of cellular or synaptic properties by a neuron or a substance released by neurons” (Katz 1999) is a topic that has received more and more attention over the last years. Formerly characterized as a form of slow and diffuse neuronal communication (Bucher and Marder 2013) is it now recognized that neuromodulation acts on multiple timescales (Nadim and Bucher 2014) and that all neuronal circuits are subject to modulatory influences (e.g., Jacob and Nienborg 2018). This modulation is easily noticeable in sensory systems where stimulus perception changes dependent on processes such as mood or attention. One of the most famous examples of such a change in perception is the “invisible gorilla experiment” (https://www.youtube.com/watch?v=vJG698U2Mvo) from Daniel Simons and Christopher Chabris who demonstrated that even large objects can become invisible if attention is directed away from them.

While evidence for neuromodulatory processes can be found across all sensory modalities (Ferezou et al. 2006; Reynolds and Chelazzi 2004; Zelano and Sobel 2005), the exact mechanism leading to changes in behavior is oftentimes hard to pinpoint, mainly for two reasons: in the immensely complex landscape of sensory processing, there are multiple neuromodulatory mechanisms for every brain area and each of those mechanisms typically also influences multiple cellular systems.

The olfactory system offers a unique opportunity for studying mechanisms underlying neuromodulatory changes in sensory systems, especially in rodents, macrosmatic animals. A major advantage of the olfactory system is its relative simplicity since primary olfactory cortices are three-layered paleocortical structures (Wilson et al. 2014) and olfactory information can reach (neo) cortical areas without being relayed via the thalamus (Moberly et al. 2018). Additionally, behavioral responses, due to changes in hormonal or nutritional status as well as attention or experience-dependent modulation, can be easily observed.

Early sensory processing, i.e., only one or two synapses downstream of primary sensory neurons, holds a special role in olfaction. The olfactory bulb (OB), the first relay station of olfactory information in the brain, is a bulbous laminar structure located anterior to the rodent forebrain. In contrast to other sensory systems where early processing structures are embedded deep inside the brain, the OB is highly accessible to physiological recording techniques. Formerly thought to represent a simple information relay between sensory input and cortex, it is now recognized as a central olfactory processing hub (Cleland 2010). This processing is highly dependent on modulatory processes originating both inside (“intrinsic neuromodulation”) as well as outside (“extrinsic neuromodulation”) of the OB (see Lizbinski and Dacks 2017). Especially extrinsic sources seem to have a large influence on OB information processing as the OB receives massive efferent input from numerous brain centers outweighing the sensory input from the nose (Shepherd 1972).

The topic of OB neuromodulation has gained more interest over the last years with many new and interesting studies shedding light on the different mechanisms. Recent reviews give a good summary of particular modulatory mechanisms (Gaudry 2018; Harvey and Heinbockel 2018; Li et al. 2020; Lizbinski and Dacks 2017; Sayin et al. 2018) but mostly focus on just one or a few of the multiple sources for neuromodulation. Building on our previous work (Brunert and Rothermel 2019; McIntyre et al. 2017), we here aim to give a more comprehensive overview of extrinsic influencers of olfactory processing in mice. In this review, we significantly expanded the chapters on each specific neuromodulatory factor with a special focus on their sources, effectors within the OB, changes in cellular output, and behavioral consequences. Furthermore, we give an outlook on potential future research topics and discuss a selection of open questions in the field of OB neuromodulation that might help to further increase interest in this challenging, but very fascinating topic.

## Neuroanatomy of the vertebrate olfactory bulb

The cellular composition and synaptic connectivity of the rodent OB are reasonably well established (for review see Burton et al. 2020; Nagayama et al. 2014; Wachowiak and Shipley 2006), a prerequisite of understanding neuromodulatory effects. We want to briefly introduce the anatomy of the OB and mention cell types that have been shown to play a role as effectors of extrinsic neuromodulation.

The OB, as the first structure of odor processing, receives olfactory information from axons of olfactory sensory neurons (OSN). Axons of these primary sensory neurons traverse the cribriform plate located between the nasal cavity and the brain and bundle together forming the outermost layer of the OB, the olfactory nerve layer. Every type of OSN expresses one out of a repertoire of approximately 1200 receptors in mice (~ 350 in humans) (Glusman et al. 2001; Nei et al. 2008). Axons of sensory neurons expressing the same type of olfactory receptor are sorted within this layer and enter the OB together (Mombaerts et al. 1996; Ressler et al. 1994) to form synapses with OB neurons in functional units called glomeruli (Shepherd et al. 2004; Sicard and Holley 1984). The layer these glomeruli form is called the glomerular layer (GL). The GL hosts several types of interneurons, most notably the periglomerular neurons (PGC), a heterogeneous group of GABAergic, and partially dopaminergic neurons, some of which receive direct input from OSNs. Additional cells are the dopaminergic and GABAergic superficial short axon cells (SA), which are characterized by the interglomerular projection of their dendrites, as well as external tufted cells (ETC), glutamatergic neurons showing spontaneous rhythmic activity (for review see (Kosaka and Kosaka 2016)). The GL merges into the external plexiform layer (EPL). In the EPL various types of interneurons can be found as well as the first type of OB output neuron, the tufted cell (TC). The mitral cell (MC), the second type of OB output neuron, is located in a thin ring-like structure within the OB, the mitral cell layer (MCL). MC and TC both project to primary olfactory cortices but show different projection patterns (Igarashi et al. 2012) as well as different properties in odor processing (Ackels et al. 2020; Economo et al. 2016; Short and Wachowiak 2019). Adjacent to the MCL, the internal plexiform (IPL) layer harbors axons from MC and TC as well as ETC axon collaterals. The GCL comprises granule cells (GC) as well as deep short axon cells (dSA). These two types of inhibitory interneurons receive strong centrifugal inputs and therefore play an essential part in OB neuromodulation.

## Different forms of neuromodulatory sources for the olfactory bulb

**Fig. 1:**
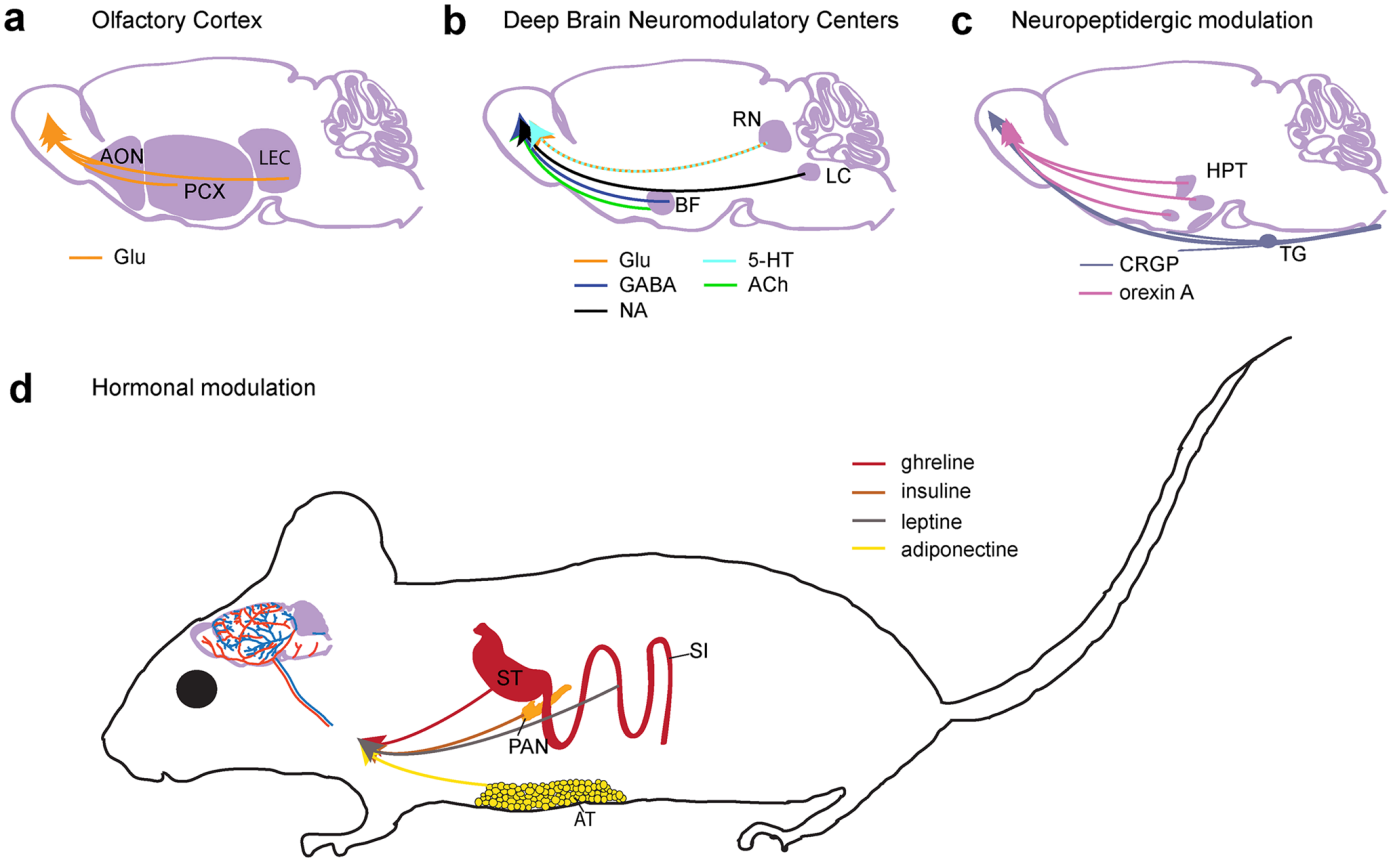
Types of extrinsic neuromodulatory inputs to the OB. Neuronal (**a**–**c**) as well as non-neuronal (**d**) sources of OB effective neuromodulatory cues. Brain-derived sources are marked in light purple while other colors mark sources outside the brain. While neuronal sources stem from fibers of brain centers projecting to the OB, non-neuronal sources secrete their cues to the bloodstream to be effective on OB receptors. (Abbreviations: AON anterior olfactory nucleus, PC piriform cortex, LEC lateral entorhinal cortex, RN raphe nuclei, LC locus coeruleus, BF basal forebrain, HPT hypothalamus, TG trigeminal ganglion, ST stomach, SI small intestine, PAN pancreas, AT adipose tissue)

Olfaction is, especially in rodents, essential for survival. Mate choice, maternal behavior, food detection, and preference as well as predator avoidance are only a few examples that critically involve the olfactory system. Thus, the plasticity and fine-tuning of olfaction to an animal’s needs are especially important. For the OB, a large number of intrinsic and extrinsic modulatory processes have been demonstrated. Extrinsic neuromodulation, i.e., modulation of olfactory processing by sources from outside the OB, can be mediated by neurotransmitters, such as GABA or glutamate, “classic” neuromodulators, like dopamine or serotonin, or by peptides, produced either by neurons (neuropeptides) or by other organs, reaching the OB via the bloodstream (hormones). Figure 1 depicts different extrinsic neuromodulation sources in the OB together with examples of the chemical messengers involved.

## Cortical top-down modulation

The primary olfactory cortex, i.e., areas with direct OB input, comprises the anterior olfactory nucleus, tenia tecta, dorsal peduncular cortex, piriform cortex, olfactory tubercle, nucleus of the lateral olfactory tract, cortical amygdala, and lateral entorhinal area (Igarashi et al. 2012; Neville and Haberly 2004; Wesson 2020).

The OB receives cortical top-down inputs from at least three olfactory cortex areas (Fig. 1a) (Matsutani and Yamamoto 2008), the anterior olfactory nucleus (AON), the piriform cortex (PC), and the lateral entorhinal cortex (LEC). The existence of centrifugal back projections from the olfactory tubercle is currently under debate (De La Rosa-Prieto et al. 2015; Gervais 1979; Heimer 1968; In ’t Zandt et al. 2019; Shafa and Meisami 1977; Zhang et al. 2017). Although these OB projections are all glutamatergic, their effects on OB circuitry are quite complex.

### *Lateral entorhinal cortex*

The LEC receives (Igarashi et al. 2012) and transfers olfactory information from the OB to the hippocampus (Steward and Scoville 1976). It is involved in the integration of olfactory information and olfactory discrimination learning (Chapuis et al. 2013; Staubli et al. 1984). OB projections arise from the layer II calbindin-positive excitatory neurons of the ventral LEC (Leitner et al. 2016). Due to its large number of afferent inputs, it has been hypothesized that feedback from LEC could provide information about the hedonic state, recent experience, and multisensory events. Additionally, given that LEC neurons are more narrowly tuned to odors than PC neurons (Xu and Wilson 2012), it might provide highly odor specific feedback to the OB (Leitner et al. 2016). So far it is unknown how LEC projections modulate odor processing in the OB but it is interesting to note that LEC to OB signaling seems to precede odor onset, and thus could potentially prepare the OB for incoming inputs (Kay et al. 1996).

### *Piriform cortex*

The PC can be separated into two parts, the anterior piriform (aPC) and the posterior piriform cortex (pPC). They receive input from different but overlapping populations of OB output cells (Igarashi et al. 2012; Nagayama et al. 2010) and code for different aspects of odor information (Wilson and Sullivan 2011). Additionally, the aPC has been shown to host a larger number of neurons projecting to the OB compared with the pPC (Padmanabhan et al. 2016) but the functional relevance of differences between aPC and pPC-derived OB projections is, as yet, unclear. PC inputs to the OB seem to activate predominantly GCs (Boyd et al. 2012; Davis and Macrides 1981; Davis et al. 1978; Pinching and Powell 1972; Price and Powell 1970) which in turn inhibit OB output neurons (Balu et al. 2007; Boyd et al. 2012; Strowbridge 2009). More recently, a visualization of top-down projections from PC into the OB (Boyd et al. 2015; Otazu et al. 2015) displayed projections to the GCL, to target GCs and dSA cells and, to a lesser extent also, to the GL, targeting PG and SA cells. Functionally probing PC-derived fiber activity, as well as effects of PC fiber activation, revealed a possible role for PC fibers in MC decorrelation (Otazu et al. 2015) and sensory gating (Boyd et al. 2012).

### *Anterior olfactory nucleus*

The AON sends the majority of cortical top-down projections to the OB (Carson 1984; Shipley and Adamek 1984). This olfactory cortex area has been implicated in a range of different functions, including serving as the first site of integrated odor percept formation and reconstructing olfactory memory traces (Aqrabawi and Kim 2020; Haberly 2001; Levinson et al. 2020), social interaction (Oettl et al. 2016; Wacker et al. 2011; Wang et al. 2020), controlling food intake (Soria-Gomez et al. 2014), and integrating activity within and between the two OBs (Esquivelzeta Rabell et al. 2017; Grobman et al 2018; Kikuta et al. 2010; Lei et al. 2006; Schoenfeld and Macrides 1984). AON-derived axons have been shown to project to multiple layers of the OB (Padmanabhan et al. 2016; Reyher et al. 1988; Wen et al. 2019) including the GCL, as well as the EPL and the MCL. Furthermore, AON projections are bilateral; i.e., the AON does not only send axons to the ipsilateral but also, via the anterior commissure, to the contralateral OB (Brunjes et al. 2005; Illig and Eudy 2009; Wen et al. 2019). The AON can be divided into two major parts, pars principalis (AONpP) and pars externa (AONpE) (Brunjes et al. 2005). AONpP sends, similar to PC, sensory-evoked feedback to the OB (Rothermel and Wachowiak 2014) but only a few studies have investigated the influence of centrifugal AONpP projections on in vivo OB circuit function. Activation of AONpP derived fibers strongly inhibits sensory signaling of olfactory output neurons both in the anesthetized (Markopoulos et al. 2012; Medinaceli Quintela et al. 2020) as well as in the awake behaving mouse (Medinaceli Quintela et al. 2020) suggesting a type of gating function. In contrast to that, AONpE corticofugal projections seem to be exclusively contralateral (Schoenfeld and Macrides 1984; Yan et al. 2008). Neurons within AONpE integrate signals from ipsilateral and contralateral OB (Kikuta et al. 2010), and their projections to the OB seem to link mirror-symmetric MCs and TC with each other (Grobman et al. 2018), possibly to achieve odor perceptual unity.

## “Neuromodulatory” projections

The term “neuromodulatory systems” refers to small neuronal pools grouped in specific nuclei in the brainstem, the mid-brain, and the basal forebrain. Through their widespread projections, neuromodulatory centers can influence many brain regions and have a powerful effect on cognitive behavior (Avery and Krichmar 2017). Neuromodulatory centers include the locus coeruleus for noradrenergic projections, the raphe nuclei for serotonergic projections, the basal forebrain for cholinergic projections, and the ventral tegmental area and substatia nigra for dopaminergic projections (Sara 2009). These centers innervate a large variety of different brain structures which themselves are often highly interconnected, thereby complicating the investigation of each of these modulatory centers on a particular circuit. Though the literature has tried to pin certain functions to each of the neurotransmitters, e.g., acetylcholine mediating attentional processes (D’Souza and Vijayaraghavan 2014; Parikh and Sarter 2008), serotonin influencing mood (Salomon and Cowan 2013), and noradrenaline being responsible for alertness (Waterhouse and Navarra 2018) it becomes more and more apparent that their function is far more complex and even direct interactions between neuromodulatory systems have to be considered (e.g., cholinergic innervation of raphe (Kalen and Wiklund 1989)).

**Fig. 2:**
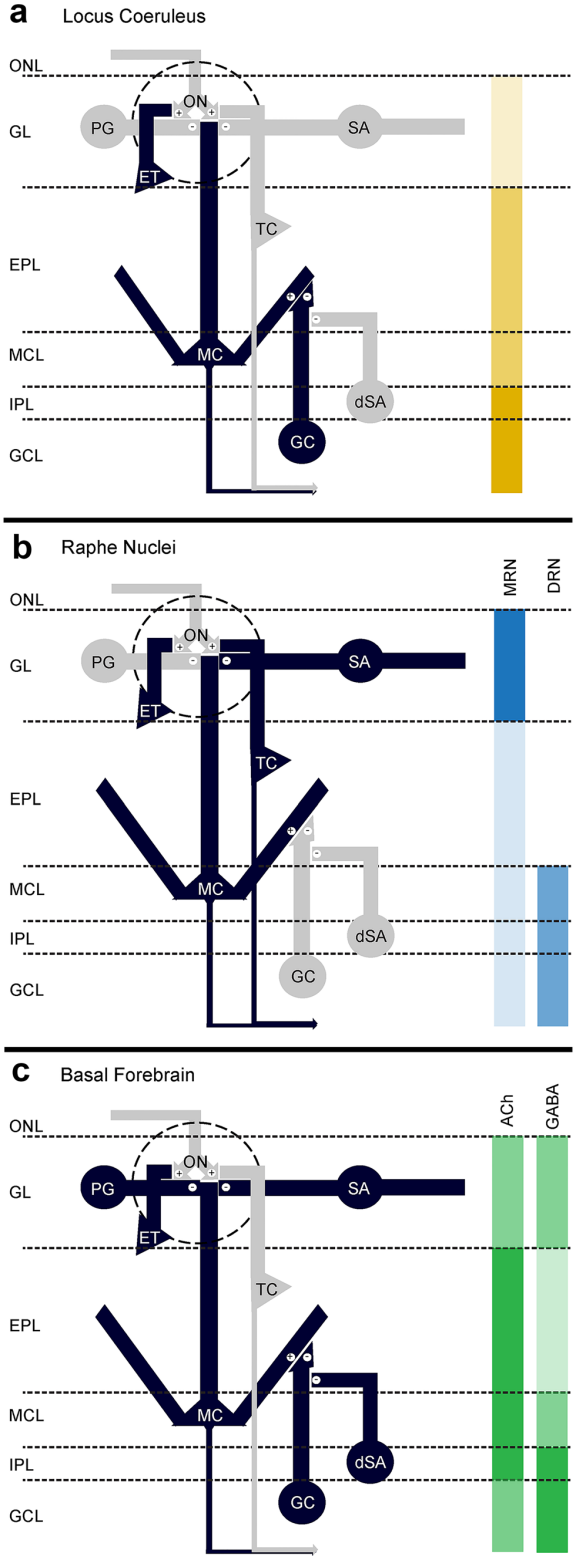
Targets of projections from neuromodulatory centers. Innervation strength and putatively affected OB cells for fibers from locus coeruleus (**a**, yellow), raphe nuclei (**b**, blue), and basal forebrain (**c**, green). Putatively affected cell types in the basal scheme of the olfactory bulb neuronal circuits are marked in black for each neuromodulatory center. Relative innervation density is marked on the right as color depth. OB modulation from BF is separated into cholinergic (ACh) and GABAergic (GABA) fibers, while innervation from raphe nuclei is separated into fibers coming from median (MRN) and dorsal raphe (DRN). (Abbreviations: ONL olfactory nerve layer, GL glomerular layer, EPL external plexiform layer, MCL mitral cell layer, IPL internal plexiform layer, GCL granule cell layer, ON olfactory nerve, PG periglomerular cells, SA short axon cells, ETC external tufted cells, MT mitral and tufted cells, GC granule cells, dSA deep short axon cells)

The OB receives centrifugal projections from at least three of these neuromodulatory systems (Fig. 1b), the locus coeruleus (LC), the basal forebrain (BF), and and the raphe nuclei. Also, a direct dopaminergic input to the OB from substatia nigra has been suggested (Hoglinger et al 2015). However, while there are clear effects of dopaminergic neuron ablation detectable in the OB circuitry (Zhang et al. 2015) and olfactory perception (Hoglinger et al. 2015), it is still unclear if this is due to a direct connection, since earlier results (Hoglinger et al. 2015) could not be confirmed by newer tracing studies (Padmanabhan et al. 2018; Schneider et al. 2020; Vinograd et al. 2019; Wen et al. 2019). Thus we will focus on the remaining three modulatory centers (Fig. 2), discussing their OB innervation, targets, cellular activity modifications, and behavioral effects.

### *Locus coeruleus*

The LC was the first neuromodulatory center characterized anatomically and neurochemically (reviewed in Chandler et al. 2019). The LC is located deep in the pons and contains only about 1500 neurons per hemisphere in rodents. Despite its small number of cells, recent studies suggest that the LC could be subdivided into different modules enabling targeted neuromodulation (Plummer et al. 2020; Uematsu et al. 2017). Immunohistochemical evidence indicates that the vast majority of LC neurons are noradrenergic (Grzanna and Molliver 1980).

The LC is the major source of forebrain noradrenaline (NA, norepinephrine) and sends projections to almost all brain regions (Sara 2009). Its activity is commonly associated with arousal (e.g., O’Hanlon 1965; reviewed in, e.g., Berridge and Waterhouse 2003: Sara and Bouret 2012). LC noradrenergic fibers reach all OB layers with the lowest and highest density in the glomerular and internal plexiform layer, respectively (Fig. 2a) (McLean et al. 1989). Consistent with this distribution, the effect of NA in the GL has received very little attention but newer results show that electrical LC stimulation elicits a global and persistent inhibition of OB input signals (Eckmeier and Shea 2014). Additionally, an excitatory role for beta-adrenergic receptors on the firing and bursting frequency of ETCs could be demonstrated (Zhou et al. 2016). Research on noradrenergic modulation of OB output initially created conflicting results with studies reporting either excitatory or inhibitory effects (Hayar et al. 2001; Jahr and Nicoll 1982; McLennan 1971; Mouly et al. 1995; Okutani et al. 1998; Perez et al. 1987; Salmoiraghi et al. 1964). More recent research was able to resolve these discrepancies and show that besides a direct excitation of MC firing through activation of the α1A-adrenergic receptor (Ciombor et al. 1999), NA also affects GCs concentration-dependently through α1A receptors, thereby increasing inhibitory drive from GCs onto MCs (Zimnik et al. 2013) and through α2A decreasing this inhibitory drive (Nai et al. 2009, 2010; Pandipati et al. 2010). The bidirectional modulation of GCs is elicited by a change in GC subthreshold membrane potential and thus excitability (Li et al. 2015; Nai et al. 2010). Further, it has been shown that LC activation reduces spontaneous MC activity while enhancing odorant responses (Manella et al. 2017). The net effect of LC-derived OB activation and inhibition was suggested as a mechanism to enhance signal-to-noise levels, a function that has been attributed to the LC in multiple sensory systems (McBurney-Lin et al. 2019). Consistent with a decrease in signal to noise-ratio, behavioral tests showed that noradrenergic modulation affects odor detection (Escanilla et al. 2012, 2010; Linster et al. 2011) and discrimination (Doucette et al. 2007; Ramirez-Gordillo et al. 2018). Additionally, the LC seems to affect OB olfactory memory encoding profoundly. The depletion of LC neurons decreased odor habituation, which could be counteracted by local bulbar NA infusions (Guerin et al. 2008). Also, reward-driven discrimination of very similar odors could be affected by NA through an interplay of α and β adrenergic receptors (Doucette et al. 2007; Mandairon et al. 2008), while new research shows an effect of NE on memory stability in the OB (Linster et al. 2020).

### *Raphe nuclei*

The serotonergic neuromodulatory system is involved in a wide range of physiological brain functions including memory, circadian rhythm, feeding, sleep-wake cycle, and stress coping (Filip and Bader 2009) and has been implicated in several neurological diseases. It represents the most diverse CNS signaling network (Grandjean et al. 2019) comprising a range of neurons displaying divergent cellular properties in terms of anatomy, morphology, hodology, electrophysiology, and gene expression (Okaty et al. 2019).

The source of all serotonergic projections is located in the brain stem in the form of nine distinct neuronal clusters (Dahlstroem and Fuxe 1964). Two of those seem to be the source of OB innervation: B7, which belongs to the dorsal raphe nucleus (DRN), and B8, which is part of the median raphe nucleus (MRN) (Muzerelle et al. 2016). Those nuclei differentially target the OB with the DRN projecting to deeper layers, specifically the MCL and GCL, while the MRN projects almost exclusively to the GL (Fig. 2b) (Muzerelle et al. 2016; Steinfeld et al. 2015). Single serotonergic fibers have been shown to possess varicosities in all layers of the OB and to form synapses with chemically heterogeneous cell populations (Suzuki et al. 2015).

The range of different serotonin receptors expressed in the OB further complicates revealing its function. At least eight of the 14 known subtypes of serotonin receptors seem to be expressed in the rodent OB (Filip and Bader 2009; Gaudry 2018), most of which have not yet been characterized functionally.

Though far from being exhaustively investigated, several modulation mechanisms for serotonin in the OB have been shown. The most prominent effect is an activation of periglomerular SAs (Brill et al. 2016; Brunert et al. 2016; Hardy et al. 2005a; Petzold et al. 2009) through 5-HT2C receptors. This leads to a reduction of presynaptic OSN activity (Petzold et al. 2009) while increasing inhibitory drive in the glomeruli. At the same time, ETCs are strongly activated, either through 5-HT 2A receptors (Brill et al. 2016; Liu et al. 2012) or glutamatergic input from raphe-derived fibers (Kapoor et al. 2016). Such dual release of glutamate and 5-HT from raphe fibers has been also shown in other brain areas like VTA (Wang et al. 2019). Output neurons can be directly activated by serotonin (Schmidt and Strowbridge 2014), in parts through 5-HT 2A receptors (Hardy et al. 2005a) or potentially also directly inhibited by 5-HT1a and/or 5-HT1b receptors (Kapoor et al. 2016). The net effect on MCs and TCs differs, while optogenetic activation of raphe-derived fibers led to TC excitation, and ultimately to a larger response correlation, MCs showed bimodal effects that led to a decorrelation of odor responses (Kapoor et al. 2016).

Despite this clear evidence for serotonin modulation of cellular activity, it is unclear how this affects olfactory behavior. So far, two studies looked at olfactory related behavior after depletion of the serotonergic system. None of them were able to establish a significant phenotype for serotonin depleted mice in different assays testing coarse olfactory performance (Carlson et al. 2016; Liu et al. 2011). Still, not only the described changes in OB cell activity but also the differentiated innervation and extensive compensatory regulation of serotonergic fiber density in the GL upon olfactory sensory deprivation (Gomez et al. 2006) make a lack of serotonergic function in the OB unlikely. More specific serotonergic targeting techniques as well as more refined olfactory tests, like discrimination of very similar odor mixtures or detection of low odor concentrations, will most likely reveal deeper insights into serotonergic function.

### *Basal forebrain*

The BF is, like the raphe, a complex of subcortical nuclei, including the medial septum, vertical and horizontal limbs of the diagonal band, the magnocellular preoptic nucleus, and the substantia innominata. BF neuromodulatory systems are thought to enhance sensory processing and amplify the signal-to-noise ratio of relevant responses (Disney et al. 2007; Goard and Dan 2009; Picciotto et al. 2012; Sarter et al. 2005) as well as being key players in mediating attentional modulation of sensory processing and coordinating cognitive operations. So far, most of these functions have been attributed to cholinergic signaling but most BF nuclei also contain GABAergic as well as glutamatergic projection neurons (Agostinelli et al. 2019; Gritti et al.^2006^; Henny and Jones 2008; Yang et al. 2017; Zaborszky et al. 2015).

The olfactory system is heavily innervated by centrifugal inputs from the BF with the majority of bulbopetal neurons located in the horizontal dorsal band of Broca (HDB) (Gielow and Zaborszky 2017; Gracia-Llanes et al. 2010; Li et al. 2018; Shipley and Adamek 1984; Zaborszky et al. 1986, 2015). So far, there is knowledge on cholinergic and GABAergic projection fibers in the OB though they seem to make up only about 50% of HDB derived projections to the OB (Zaborszky et al. 1986). Cholinergic and GABAergic bulbopetal cells show an overlapping but largely segregated pattern in BF (Zaborszky et al. 1986) and innervate the OB layers differently (Bohm et al. 2020). ChAT-positive cholinergic axon terminals are visible in all layers of the OB (Fig. 2c) (Bohm et al. 2020; Durand et al. 1998; Gomez et al. 2005; Macrides et al. 1981; Rothermel et al. 2014; Salcedo et al. 2011) but innervate the superficial OB layers rather homogeneously compared with the GCL, which receives less input. Glutamate decarboxylase (GAD) 2-positive GABAergic axon terminals, in contrast, show a strong innervation of the glomerular and the GCL with weaker innervation of EPL and MCL (Fig. 2c) (Bohm et al. 2020: Nunez-Parra et al. 2013). Fine structural observations show that cholinergic projections synapse primarily onto interneurons (Kasa et al. 1995; Nickell and Shipley 1988) but extrasynaptic transmission is, like for all neuromodulatory transmitters, a well-known feature (Fuxe et al. 2012).

In the OB nicotinic as well as muscarinic acetylcholine receptors are expressed. So far nicotinic acetylcholine receptor (nAChR) subunits α2, α3, α4, α5, α6, α7, and α9 (Keiger and Walker 2000) as well as muscarinic acetylcholine receptors (mAChRs) M1 and M2 (Le Jeune et al. 1995) have been detected. mAChRs are present primarily in the EPL and GL (Hunt and Schmidt 1978; Le Jeune et al. 1995) and while M1 activation enhances GC excitation, leading to suppression of MTC excitability (Pressler et al. 2007; Smith and Araneda 2010), M2 seems to exert its function in the GL (Bendahmane et al. 2016). Effects have varied from excitation of MTC glomerular responses (Bendahmane et al. 2016) to an inhibition of MC and ETC spiking due to activation of inhibitory interneurons (Liu et al. 2015). nAChRs have mainly been found in the GL and MCL (Hunt and Schmidt 1978; Le Jeune et al. 1995). So far, functional nAChRs have been located on MCs and ETCs (D’Souza et al. 2013; D’Souza and Vijayaraghavan 2012) but recent research also suggests α2 subunit-containing nAChRs on dSAs (Burton et al. 2017; Case et al. 2017). Studies on nAChR signaling in the OB have focused mainly on receptors on ETCs and MCs and found that ACh application or optogenetic OB cholinergic fiber activation in slices leads to a direct MC and ETC activation (D’Souza et al. 2013; D’Souza and Vijayaraghavan 2012; Liu et al. 2015). Overall, observed ACh effects on OB odor processing reach from input independent sensory gain modulation (Bohm et al. 2020; Rothermel et al. 2014) similar to reports in visual cortex (D’Souza et al. 2013; D’Souza and Vijayaraghavan 2012; Parsa et al. 2015) to low pass filtering (Bendahmane et al. 2016) and sharpening of receptive fields (Ma and Luo 2012).

The role of BF-derived GABAergic fibers in the OB has been much less investigated. GABAergic fibers from HDB and the magnocellular preoptic nucleus have been shown to influence GCs (Nunez-Parra et al. 2013), dSAs (Case et al. 2017), and different PGCs (Sanz Diez et al. 2019). A recent study reported that optogenetic activation of GABAergic fibers in the OB caused inhibition of spontaneous and weak sensory activity while increasing odor-evoked responses (Bohm et al. 2020). This suggests a function for the GABAergic BF system in the modulation of signal-to-noise ratio or high pass filtering weak sensory inputs. Little is known on the behavioral effects of GABAergic OB modulation except that pharmacological inactivation of GABAergic fibers impairs olfactory sensitivity (Nunez-Parra et al. 2013). In contrast, cholinergic BF fibers have been shown to improve odor discrimination ability (Chaudhury et al. 2009; Cleland et al. 2002; Doty et al. 1999; Li and Cleland 2013; Mandairon et al. 2006) as well as to facilitate olfactory learning and memory (Devore and Linster 2012; Ravel et al. 1994; Ross et al. 2019). Future studies will shed light on the function and interplay of the cholinergic and GABAergic BF system, how it influences OB odor processing, and its impact on olfactory guided behavior.

## Neuropeptidergic modulation

A large number of neuropeptides can modulate OB function, and most of them are generated locally within the OB, e.g., somatostatin (SOM; Nocera et al. 2019), glucagon-like peptide 1 (GLP-1; Thiebaud et al. 2019), pituitary adenylate cyclase-activating polypeptide (PACAP; Irwin et al. 2015), or the circadian rhythm mediating vasoactive intestinal polypeptide (VIP; Lukas et al. 2019; Miller et al. 2014). Since some neuropeptides, like substance P or enkephalins, are located both in local cells and in axonal fibers in the OB (Halasz and Shepherd 1983), effects cannot be assigned to extrinsic or intrinsic sources. Additionally, there are neuropeptides found exclusively in secretory fibers from other neuronal centers that project to the OB (Fig. 1c), like, e.g., calcitonin gene-related peptide (CGRP)–containing fibers from the trigeminal ganglion. These fibers were reported to reduce the activity of OB interneurons, thus mediating interaction between trigeminal and odorant sensations (Genovese et al. 2016).

Another example is orexin-A. Orexin is a neuropeptide involved in sleep/wake regulation (Sakurai et al. 2010) as well as feeding behavior (Horvath and Gao 2005; Sakurai et al. 1998). Orexin-positive fibers from the lateral hypothalamus have been shown in the OB (Gascuel et al. 2012; Peyron et al. 1998) with varicose fibers located predominantly in the GL, MCL, and GCL (Caillol et al. 2003). Orexin receptors 1 and 2 are expressed in PGCs, MCs/TCs, and GCs, and orexin-A was shown to directly activate and indirectly inhibit MC activity (Hardy et al. 2005b) suggesting an additional way of metabolic regulation of olfactory processing.

Another neuropeptide that has received attention in recent years is oxytocin (OTX), which controls childbirth and is strongly involved in social behaviors. OTX released in the forebrain mainly originates from neurons in the paraventricular nucleus of the hypothalamus (Knobloch et al. 2012). Recently, it has been reported that same-sex social recognition in mice is OTX dependent (Linster and Kelsch 2019, Oettl et al. 2016). OTX was shown to activate AON cells projecting to the OB thereby modulating MC firing (Oettl et al. 2016). Together with the absence of detectable OTX fibers in the OB (Knobloch et al. 2012), this is casting doubt on a direct effect of OTX in the OB. However, the weak but clear presence of oxytocin receptors in the OB (Ferguson et al. 2000; Ferris et al. 2015; Vaccari et al. 1998), the effects of OTX infused into the OB on maternal behavior (Yu et al. 1996a), and MC firing (Yu et al. 1996b), as well as the fact that expression of oxytocin and its receptors is highly regulated (Freund-Mercier et al. 1994), open up the question of a to-date unknown or just undetected function of OTX for the OB.

## Hormonal neuromodulation

Despite its importance for mating and nutrition, hormonal neuromodulation is a field that has received less attention. The OB is well-positioned for hormonal neuromodulation; certain blood molecules can reach the OB more easily compared with other brain areas since the density of the blood capillary network, especially in the GL, is very high (Lecoq et al. 2009) and the blood-brain barrier at the OB is more permeable (Ueno et al. 1996). A specialized transport system for certain hormones provides an additional means to increase the local concentration of those hormones within the OB (Banks et al. 1999).

Hormones have many diverse functions, e.g., sex steroids like testosterone or estradiol, that regulate sexual differentiation and behavior (McEwen and Milner 2017); neurohormones like melatonin, which affects circadian rhythms (Brown 1994); and metabolic hormones like ghrelin and insulin (Julliard et al. 2017). Receptors for both estrogens (Hoyk et al. 2014, Maruska and Fernald 2010) and melatonin (Corthell et al. 2014) are expressed in the OB, and hormonal effects could be demonstrated (Corthell et al. 2014; Dillon et al. 2013). However, the presence of synthesizing enzymes for these hormones within the OB (Corthell et al. 2014; Hoyk et al. 2014) speaks rather for a local neuropeptidergic function.

Remotely produced hormones that act on OB cells have so far been linked to the metabolic regulation of food intake (see (Palouzier-Paulignan et al. 2012). The olfactory system is known for its major contribution to the hedonic evaluation of food (with effects on food choices and consumption), and it seems to make sense that olfaction would be modulated according to foraging needs (Julliard et al. 2017). Foraging influencing hormones are divided into orexigenic (appetite-stimulating) and anorexigenic (appetite-suppressing) hormones. So far, ghrelin and adiponectin as orexigenic molecules and insulin and leptin as anorexigenic molecules have been identified. These hormones have different sources (Fig. 1d): ghrelin is produced primarily by the stomach (Kojima et al. 1999), leptin is predominantly generated by adipose cells and enterocytes in the small intestine (Bado et al. 1998), adiponectin is synthesized predominantly in adipose tissue (Scherer et al. 1995), while insulin is released by pancreatic beta cells in response to feeding state in a glucose-dependent manner (Henquin 2011).

The best-investigated metabolic hormone with a function in the OB is insulin. The OB shows the highest insulin receptor (insulin kinase) density in the whole brain (Hill et al. 1986) and insulin has been shown to cause an increase in firing frequency and inhibition of spike adaptation in OB MCs (Fadool et al. 2000). As a substrate, the voltage-activated K+ channel Kv1.3 has been identified which, when phosphorylated by insulin receptor kinase, is causing a change in MC excitability (Fadool et al. 2011). Adiponectine receptors have been found in all OB cell layers, and OB adiponectine injection was found to regulate the expression of insulin receptors (Miranda-Martinez et al. 2017).

Ghrelin is transported across the blood-brain barrier and is present in high concentrations in the OB (Rhea et al. 2018). So far only one ghrelin receptor has been identified, growth hormone secretagogue receptor (GHSR-1a) which is expressed in GL and MCL (Tong et al. 2011). Functionally, ghrelin has been shown to increase exploratory sniffing behavior and olfactory sensitivity but it is unclear whether this effect is due to local ghrelin signaling.

The OB has also high levels of leptin receptors (Shioda et al. 1998) but despite studies showing leptin decreasing olfactory sensitivity (Julliard et al. 2007) and an increase in performance of leptin-deficient mice in olfactory detection (Getchell et al. 2006) and memory tasks (Chelminski et al. 2017), the cellular mechanisms of these changes remained unclear for a long time. Only recently it was shown that leptin decreases the excitability of MCs/TCs as well as GCs through direct modulation of a voltage‐sensitive potassium channel which leads to a net inhibition of the MTC population and negatively affects discrimination performance (Sun et al. 2019).

As mentioned for ghrelin, it is not exactly clear if the orexigenic and anorexigenic effects of the hormones are caused by their effects in OB circuits or if changing the sense of smell is a secondary effect. Global developments of increased obesity and subsequent research in diet and metabolism will shed more light on this relationship.

## Open questions concerning neuromodulation in the olfactory bulb

There are many unresolved questions in the field of olfactory neuromodulation; maybe the most prominent being when and how modulatory processes are used in olfactory behavior. An important step in this direction is defining the nature of OB projection neurons. New tracing techniques are not only able to label cells in higher brain areas according to their postsynaptic targets but also allow for a defined characterization of their inputs (Schwarz et al. 2015). First results defining the input-output relations of different brain centers indicate that there are great differences between neuromodulatory systems, with, e.g., the LC rather resembling a homogenous integrator and broadcaster of information (Schwarz et al. 2015), while input-output relations in the BF seem to be much more specific (Gielow and Zaborszky 2017). Other technical advances in, e.g., the development of faster, more sensitive optogenetic tools for cell type-specific dissection of brain circuits (Lee et al. 2020), increasing spatial resolution for deep brain imaging (Vasquez-Lopez et al. 2018), and enhancing sensitivity and expression of genetically coded calcium dyes (Dana et al. 2019) will help to advance our knowledge on the specific functions of different neuromodulatory systems in olfactory guided behaviors.

Another topic that bears consideration are the numerous interactions between different brain areas providing modulatory input to the OB. These interactions can occur outside of the OB but also influence their input *to* and neuromodulation *in* the OB. The AON for example sends odor specific feedback to the OB but also receives input from olfactory cortical areas like the aPC (Haberly 2001; Haberly and Price 1978; Luskin and Price 1983) as well as non-olfactory areas such as BF (Broadwell and Jacobowitz 1976; Carnes et al. 1990; De Carlos et al. 1989; Gaykema et al. 1990; Luiten et al. 1987; Zaborszky et al. 2012). The AON has also been implicated as the mediator of hypothalamic oxytocin effects on OB olfactory processing (Oettl et al. 2016). However, such interactions can also occur inside the OB where ACh (Zhou et al. 2018), endocannabinoids (Pouille and Schoppa 2018), and GABA (Mazo et al. 2016) have been shown to modulate synapses between corticofugal fibers and cells of the OB.

A huge step forward would also be the detailed characterization of projecting neurons in terms of transmitter release inside the OB. The here described BF, which has been classically associated with cholinergic modulation, is a good example. Cholinergic and GABAergic neurons account only for about 50% of all BF-derived bulbopetal neurons (Zaborszky et al. 1986) leaving open the functional contribution of the remaining half. Additionally, most bulbopetal fibers are at least suspected to contain more than one transmitter. Glutamatergic effects upon stimulation of serotonergic OB fibers (Kapoor et al. 2016) and GABA and ACh corelease by a subpopulation of OB projecting HDB neurons are just two examples (Case et al. 2017).

Another interesting topic is the plasticity of neuromodulatory systems. Apart from developmental changes in the embryonic phase, top-down systems are also highly plastic in postnatal mice. For example, sensory deprivation through unilateral naris occlusion was shown to change cholinergic innervation patterns in the OB (Salcedo et al. 2011) though overall fiber density remained unchanged (Gomez et al. 2006; Salcedo et al. 2011). Noradrenergic OB input is even more plastic and shows a strong change in LC derived fiber density (Gomez et al. 2006), as well as adrenergic receptor expression upon reduced sensory input. Additionally, neuromodulatory effects are not homogenously distributed across different OB glomeruli. Both, serotonergic (Gomez et al. 2005) and cholinergic fibers (Gomez et al. 2005; Macrides et al. 1981; Salcedo et al. 2011), have been shown to innervate some glomeruli stronger than others but the functional significance is unknown.

**Fig. 3:**
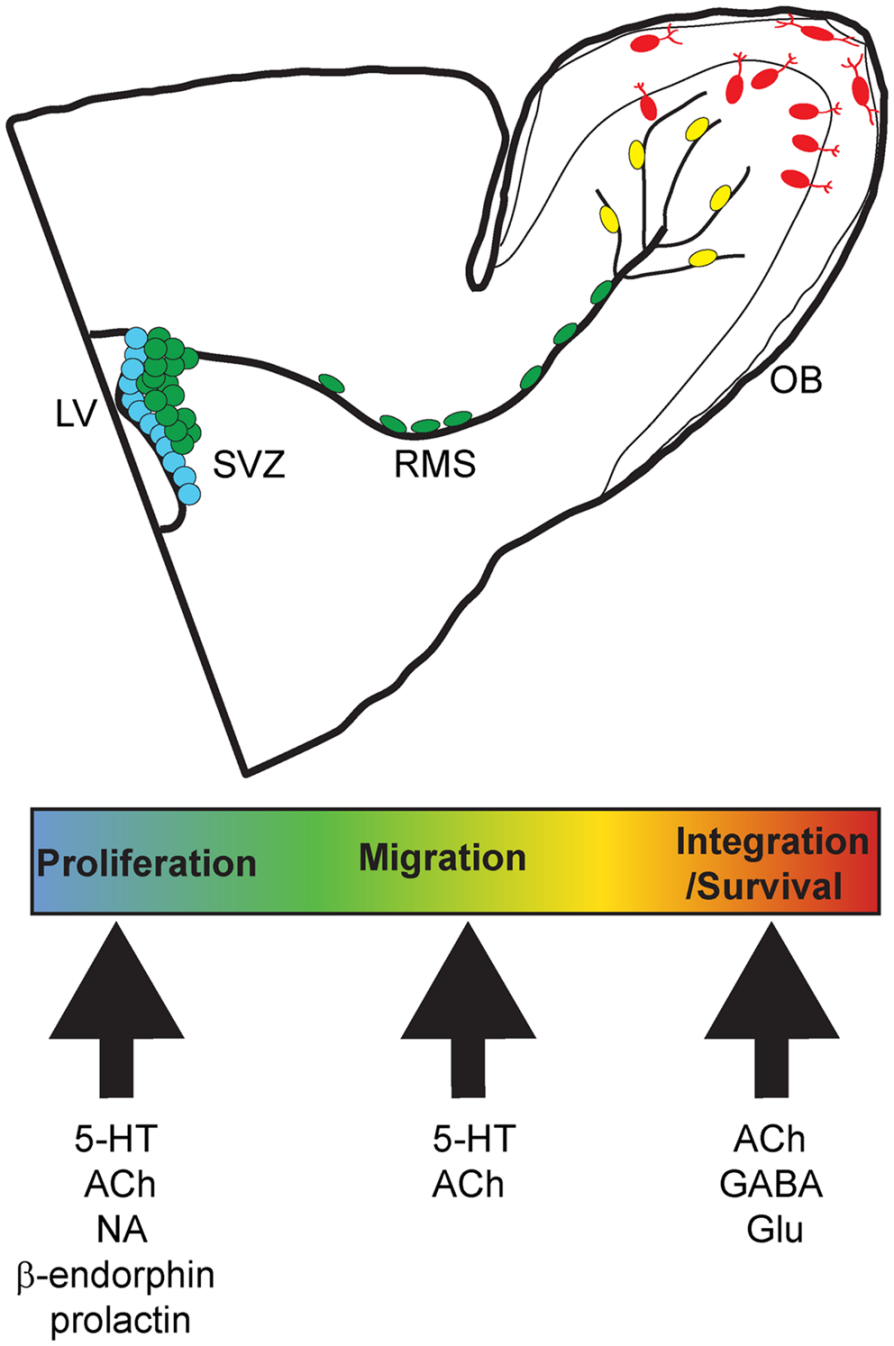
Neuromodulatory influences in adult neurogenesis (adapted from (Rikani et al. 2013). Neuromodulatory transmitters coming from deep brain neuromodulatory centers, olfactory cortex, or hypothalamus can influence olfactory processing not only by immediate effects but also by acting on proliferation (blue), migration (tangential (green), radial (yellow)), or differentiation and survival (red) of adult-born neurons. Neuromodulators acting on a specific process are listed under the respective arrows. (Abbreviations: OB olfactory bulb, RMS rostral migratory stream, SVZ subventricular zone, LV lateral ventricle)

Finally, one question that has received more interest of late is adult neurogenesis. A distinguishing feature of the OB is its lifelong integration of adult-born neuronal progenitors into its inhibitory circuits. OB neurogenesis has been shown as an important factor for olfactory processing (Livneh et al. 2014), odor discrimination (Gheusi et al. 2000; Mouret et al. 2009), and odor learning (Alonso et al. 2012; Lazarini et al. 2009; Sultan et al. 2010). Several recent reviews stress the importance of adult neurogenesis for olfactory function (Hanson et al. 2017; Lledo and Valley 2016; Takahashi et al. 2018). Neuronal progenitors for adult OB neurogenesis stem from the subventricular zone (SVZ) along the walls of the brain’s lateral ventricle (Merkle et al. 2007) where a pool of dividing astrocytes constantly produces new neuroblasts (Doetsch et al. 1999). From the SVZ neuroblasts migrate tangentially along the rostral migratory stream to the core of the OB, then radially to the superficial GCL and, to a lesser extent, the GL (Lepousez et al. 2013; Lledo et al. 2006). It has been estimated that in young adult rodents 10,000–30,000 neuroblasts per day reach the OB (Lois and Alvarez-Buylla, 1994); however, only 50% of those cells survive for more than a month (Petreanu and Alvarez-Buylla 2002), suggesting a rigorous selection process. Numerous studies have shown that new interneurons can be influenced in all phases of their generation, migration, and integration and that this influence is mediated by multiple neuronal messengers from different sources (see Fig. 3). For example, all classical neuromodulators seem to influence adult neurogenesis like, e.g., acetylcholine, either from local sources (Paez-Gonzalez et al. 2014) or BF derived fibers, regulates the proliferation, migration, and survival of adult-born neurons (Kaneko et al. 2006; Mechawar et al. 2004; Paez-Gonzalez et al. 2014; Sharma 2013). Additionally, new research has shown that HDB GABAergic fibers target immature GCs upon arrival in the OB and promote their survival (Hanson et al. 2020). Other neuromodulators involved are 5-HT that acts on proliferation and migration (Banasr et al. 2004; Garcia-Gonzalez et al. 2017), dopamine that increases proliferation (Hoglinger et al. 2004), and norepinephrine (Weselek et al. 2020) acting on proliferation in the SVZ. Other modulatory influences on OB neurogenesis come from cortical fibers that reach into the GCL and establish synaptic connections to newborn neurons (Arenkiel et al. 2011; De La Rosa-Prieto et al. 2015; Deshpande et al. 2013). Activation of these fibers can induce LTP (Nissant et al. 2009) as well as experience-dependent plasticity (Lepousez et al. 2014) and thus seems to promote the survival of these neurons. Other examples are neuropeptides like prolactin from the pituitary gland during pregnancy (Shingo et al. 2003) or β-endorphin from hypothalamic neurons in hunger and satiety (Paul et al. 2017). Taken together neuromodulatory changes in neurogenesis will be of great interest in the future, especially since a recent paper demonstrated that cholinergic effects on olfactory learning require adult neurogenesis (Schilit Nitenson et al. 2019).

## Conclusion

In this review, we have aimed to give a broad overview on extrinsic neuromodulation of the OB. The discussed list of external OB neuromodulatory sources is, however, by no means complete. Increasingly sensitive tracing techniques are already expanding the list of centrifugal inputs to the OB (Padmanabhan et al. 2018; Schneider et al. 2020; Wen et al. 2019) and studies on transcriptomes of OB cells might also increase knowledge on hormonal receptors.

In summary, we can say that although most bulbopetal connections have been characterized more than 40 years ago, we are still far away from getting a comprehensive view of processes that lead to modulation of early olfactory processing. Given the intense role of neuromodulation in neurological diseases (see, e.g., Avery and Krichmar 2017), more research is needed in this field.
